# Tremor and Movement Slowness Are Two Unrelated Adverse Effects Induced by Valproate Intake

**DOI:** 10.1002/mdc3.13560

**Published:** 2022-09-25

**Authors:** Alessandro De Biase, Giulia Paparella, Luca Angelini, Antonio Cannavacciuolo, Donato Colella, Emanuele Cerulli Irelli, Anna Teresa Giallonardo, Carlo Di Bonaventura, Alfredo Berardelli, Matteo Bologna

**Affiliations:** ^1^ Department of Human Neurosciences Sapienza University of Rome Rome Italy; ^2^ IRCCS Neuromed Pozzilli (IS) Italy

**Keywords:** valproate‐induced tremor, movement slowness, kinematic finger tapping, bradykinesia, cerebellum

## Abstract

**Background:**

To date, only a few clinical and neurophysiological studies have assessed the features of valproate‐induced tremor (VIT), and whether valproate (VPA) affects voluntary movements is underinvestigated.

**Objective:**

To better characterize the clinical and neurophysiological features of VIT in patients with epilepsy and the effect of VPA on the execution of voluntary movement.

**Methods:**

We tested 29 patients with VIT (13 taking VPA alone and 16 taking VPA plus other antiepileptics). Patients underwent a neurological examination, video recordings and kinematic assessments of postural, kinetic, and resting upper limb tremor using a motion analysis system. Movement execution was tested by kinematic assessment of finger tapping. Data of patients with VIT were compared with those of 13 patients with epilepsy taking VPA but without tremor, 13 patients with epilepsy who were not on VPA treatment, 20 patients with Parkinson's disease (PD), and 20 healthy controls (HCs).

**Results:**

Clinical and kinematic evaluations showed that tremor in patients taking VPA alone was less severe than tremor in patients taking VPA plus other antiepileptics. All patients taking VPA, regardless of the presence of tremor, performed slower finger tapping compared with HCs, similar to what was observed in PD, although with no sequence effect. Patients with epilepsy without VPA showed a normal motor performance.

**Conclusions:**

Tremor and movement slowness are motor signs induced by VPA. VIT severity is exacerbated when VPA is taken in combination with other antiepileptics. VPA‐induced slowness occurs regardless of tremor, may precede tremor development, and is not attributed to epilepsy.

Valproate (VPA) is one of the most effective and widely used antiepileptic drugs. However, clinical studies have reported that up to 45% of patients taking VPA may develop tremor,^1^, ^2^, ^3^ and neurophysiological investigations found that VPA‐induced tremor (VIT) can be present in up to 80% of patients.^4^, ^5^, ^6^, ^7^, ^8^ VIT is a postural and kinetic tremor of the upper limbs. Dose‐dependent severity and mild progression over time have also been described in VIT.^4^, ^9^ From a neurophysiological standpoint, VIT is characterized by a low amplitude and high frequency, ranging from 6 to 15 Hz, thus resembling essential tremor (ET).^4^, ^5^, ^10^ Recently, we used kinematic methods to investigate tremor characteristics in patients with VIT compared with patients with ET.^8^ We confirmed that postural and kinetic upper limb tremor were the main features in patients with VIT. We also found that VIT frequently occurred in the voice, head, and lower limbs. Moreover, we demonstrated a high occurrence of rest tremor in VIT as compared with ET.^8^ However, some of the patients we previously studied were taking VPA plus other antiepileptic drugs that have been demonstrated to induce or exacerbate tremor,^9^ and we did not specifically assess the possible influence of other associated treatments on VIT features and severity.^8^ In this regard, the observation that other antiepileptics may exacerbate tremor in patients taking VPA is mainly based on clinical case reports and series,^11^, ^12^ which have not always reported consistent results,^3^, ^13^ and is not supported by neurophysiological evidence.

In addition to tremor, VPA may also cause parkinsonism, including bradykinesia and other signs.^2^, ^3^, ^14^, ^15^ In our previous study on VIT, we found that patients with VIT were slower than patients with ET when performing upper limb movements.^8^ However, we could not rule out the influence of tremor on upper limb motor execution, that is, movement slowness might have represented an attempt to minimize the detrimental effect of kinetic tremor on motor arm performance in these patients. To our knowledge, only one previous study investigated possible finger and hand repetitive movement abnormalities in patients taking VPA with tremor (tVPA) by using quantitative methods.^16^ However, the relationship between tremor and altered voluntary motor performance in patients with VPA remains unclear.

In the present study, we aimed to further investigate VIT features, particularly the possible effects of other associated antiepileptic drugs on tremor features. Second, we investigated the influence of VPA on voluntary movement execution in patients as well as its association with tremor. Hence, we clinically and kinematically assessed finger tapping in patients with tVPA compared with patients with epilepsy taking VPA but without tremor (ntVPA), patients with epilepsy who were not on VPA treatment and who had never been treated with VPA (noVPA), patients with Parkinson's disease (PD),^17^, ^18^ and healthy controls (HCs). We specifically assessed finger tapping because this is the pivotal test for bradykinesia detection in clinical practice.^17^, ^18^, ^19^, ^20^, ^21^, ^22^

## Methods

### Participants

A total of 29 patients with tVPA were enrolled in the study (Table 1). Patients were consecutively recruited at the epilepsy outpatient clinic of the Department of Human Neurosciences, Sapienza University of Rome. Of 29 patients, 13 were taking VPA alone (tVPA monotherapy), whereas 16 patients were taking other antiepileptics in addition to VPA (tVPA polytherapy). Patients had a diagnosis of epilepsy defined according to International League Against Epilepsy criteria^23^ (Table 1). All patients were on VPA therapy for at least one year prior to study enrollment. In all cases of tVPA, tremor onset had started after VPA administration, with no prior tremor manifestations. No patients had a history of other neurological conditions characterized by tremor, and none were taking any other medication for tremor treatment.^9^

**TABLE 1:** Diagnosis and therapy of patients with epilepsy

Subject	Diagnosis	Treatment and Daily Dose
	**tVPA monotherapy**	
1	IGE	VPA 1000 mg
2	IGE	VPA 1000 mg
3	IGE	VPA 1000 mg
4	IGE	VPA 1000 mg
5	IGE	VPA 1000 mg
6	IGE	VPA 1200 mg
7	IGE	VPA 1000 mg
8	IGE	VPA 1000 mg
9	IGE	VPA 1000 mg
10	IGE	VPA 500 mg
11	FE (structural origin)	VPA 1000 mg
12	IGE	VPA 800 mg
13	IGE	VPA 800 mg
	**tVPA polytherapy**	
1	FE (unknown etiology)	VPA 1000 mg; LTG 300 mg; ZNS 200 mg
2	IGE	VPA 1000 mg; LTG 400 mg
3	IGE	VPA 1500 mg; LTG 200 mg
4	IGE	VPA 1500 mg; LTG 150 mg
5	IGE	VPA 1500 mg; ETS 1250 mg; FNB 25 mg
6	IGE	VPA 2000 mg; LTG 300 mg; LEV 3000 mg; FNB 25 mg
7	IGE	VPA 1200 mg; LCS 150 mg; LTG 200 mg; clobazam
8	FE (structural origin)	VPA 1000 mg; LCS 300 mg; LEV 2000 mg;
9	IGE	VPA 1000 mg; LTG 350 mg; TPM 200 mg
10	IGE	VPA 2000 mg; LTG 150 mg; RFM 3600 mg
11	IGE	VPA 1500 mg; LEV 500 mg
12	IGE	VPA 1500 mg; LTG 150 mg
13	IGE	VPA 1500 mg; LEV 2000 mg; PER 10 mg
14	FE (unknown etiology)	VPA 1000 mg; LCS 400 mg; CBZ 40 mg
15	IGE	VPA 1500 mg; LTG 100 mg; RFM 600 mg
16	FE (unknown etiology)	VPA 1000 mg; CBZ 20 mg; LCS 200 mg; LEV 2000 mg
	**ntVPA**	
1	IGE	VPA 800 mg
2	IGE	VPA 600 mg
3	IGE	VPA 500 mg
4	IGE	VPA 400 mg
5	IGE	VPA 1000 mg
6	IGE	VPA 1000 mg
7	IGE	VPA 1000 mg
8	IGE	VPA 1000 mg
9	IGE	VPA 900 mg
10	IGE	VPA 1000 mg
11	IGE	VPA 500 mg
12	IGE	VPA 1000 mg
13	IGE	VPA 500 mg
	**noVPA**	
1	FE (structural origin)	LEV 1000 mg
2	IGE	LEV 2500 mg
3	IGE	LEV 1000 mg
4	IGE	LEV 1000 mg
5	IGE	LEV 3000 mg
6	IGE	LEV 2000 mg
7	IGE	LTG 400 mg
8	IGE	LEV 1000 mg
9	IGE	LEV 2000 mg
10	IGE	LEV 3000 mg
11	IGE	LTG 400 mg
12	IGE	LTG 200 mg
13	IGE	LEV 3000 mg

Abbreviations: tVPA, VPA and tremor; VPA, valproate; FE, focal epilepsy; IGE, idiopathic generalized epilepsy; LTG, lamotrigine; ZNS, zonisamide; ETS, etosuccimide; FNB, phenobarbital; LEV, levetiracetam; LCS, lacosamide; TMP, topiramate; RFM, rufinamide; CBZ, carbamazepine; ntVPA, VPA without tremor; noVPA, patients with epilepsy who were not taking nor had never taken VPA.

With the aim of investigating movement execution in patients taking VPA, we also consecutively recruited 13 ntVPA patients, 13 noVPA patients, 20 patients with PD, and 20 HCs (Table 1). Of the noVPA patients, 10 were taking levetiracetam (mean daily dose ± standard deviation [SD]: 1950 ± 895.97 mg), 3 were taking lamotrigine (mean daily dose ± SD: 333.33 ± 115.47 mg). All patients with PD were diagnosed according to current clinical criteria.^17^, ^18^ The mean disease duration ± SD in patients with PD was 3.02 ± 2.66 years. The mean Hoehn and Yahr score^24^ ± SD was 1.58 ± 0.46. All patients with PD discontinued their dopaminergic treatment at least 12 hours before evaluation (mean levodopa equivalent daily dose ± SD in patients with PD: 234.05 ± 146.81). Other type of medications, including proton‐pump inhibitors, antihypertensive drugs, antiplatelet therapies, thyroid medications, and statins, were not discontinued in all participants.

Demographic and clinical data collection in participants included the following: sex, age, comorbidities, familial history, tremor onset and duration, and concomitant antiepileptic treatment. Possible cognitive and psychiatric disturbances were evaluated by means of the Montreal Cognitive Assessment,^25^ Frontal Assessment Battery,^26^ and Beck Depression Inventory.^27^ Tremor was clinically assessed using the Fahn–Tolosa–Marin Tremor Rating Scale (FTMTRS).^28^ Bradykinesia was clinically evaluated with the Movement Disorder Society–sponsored revision of the Unified Parkinson's Disease Rating Scale, Part III (MDS‐UPDRS III).^20^, ^21^ Fatigue was assessed with the Fatigue Severity Scale.^29^

All participants provided written informed consent to participate in the study. Experimental procedures were approved by the local ethics committee and performed according to the Declaration of Helsinki.

### Tremor Kinematic Recordings and Analysis

Tremor recordings were performed with a three‐dimensional (3D) optoelectronic system (SMART motion system, BTS Engineering, Milan, Italy),^8^, ^30^, ^31^, ^32^, ^33^ which includes 3 infrared cameras (120 Hz frequency) that detect motion in the 3D space of reflective markers taped to various body segments. Upper limb tremor was recorded using 4 markers placed on the distal phalange of the index finger and on the second metacarpal bone of each hand. To exclude possible contamination of trunk movements, 3 markers were also placed over the trunk.^8^, ^30^, ^31^, ^32^, ^33^ Upper limb postural tremor was recorded in 2 positions: (1) with arms outstretched in front of the chest (posture 1) and (2) with arms flexed at the elbows, that is, lateral “wing beating” posture (posture 2).^8^, ^30^, ^31^, ^32^, ^33^ Upper limb kinetic tremor was recorded during a “pointing task” in which patients repetitively moved their index finger from their nose to a reflective target fixed on a heavy support approximatively 15 cm above a table at sternal height and placed approximately at a distance equal to 2/3 of the length of the arm.^8^, ^30^, ^31^, ^32^, ^33^ Finally, upper limb rest tremor was recorded while patients sat comfortably on a chair facing the cameras, with their arms lying on a desk in front of them.^8^, ^31^ We carefully controlled that patients were fully at rest. Three 45‐second trials were recorded for each posture; three 15‐second trials were recorded during the “pointing task.”^8^, ^30^, ^31^, ^32^, ^33^

For tremor analysis, power spectra were calculated by fast Fourier transformation using dedicated software (SMART Analyzer, BTS Engineering). For postural and rest tremor analyses, the marker placed on the second metacarpal bone was considered the reference marker.^8^, ^30^, ^31^, ^32^, ^33^ We determined the magnitude of postural and rest tremor by measuring the root mean square of the acceleration traces (GRMS^2^) of the reference marker in 3D space. We then measured the dominant frequency peak (Hz) of postural and rest tremor.^8^, ^30^, ^31^, ^32^, ^33^ For kinetic tremor analysis, the marker placed on the last phalange of the index finger was considered the reference marker. A different algorithm was used to measure kinetic tremor of the upper limb.^8^, ^30^, ^31^, ^32^, ^33^ We considered the number of movements and the distance (meters) covered during the “pointing task.” We also considered the velocity and acceleration peaks (m/s and m/s^2^, respectively). The duration of the acceleration and deceleration phases, the deceleration/acceleration ratio (D/A), and the curvature index (CI), that is, arm end point average path length/length of a straight line joining the initial and final positions, were considered measures of movement quality, that is, trajectory homogeneity.^8^, ^30^, ^31^, ^32^, ^33^

### Finger Tapping Kinematic Recordings and Analysis

Kinematic assessment of voluntary movement execution was performed in all participants during repetitive finger tapping.^32^, ^34^, ^35^, ^36^, ^37^, ^38^ Reflective markers were taped on the hand and on the distal phalanx of the index finger and thumb.^32^, ^34^, ^35^, ^36^, ^37^, ^38^ Three 15‐second repetitive finger movements were recorded in succession with a 60‐second pause between each recording, that is, opening and closing the index finger and the thumb as fast and broadly as possible. Because no significant effect of handedness on motor performance has been demonstrated, we recorded finger tapping from the dominant hand in all patients with epilepsy and in HCs and from the most affected side in PD.^32^, ^34^, ^35^, ^36^, ^37^, ^38^ For the analysis, we considered the total number of movements and the movement rhythm, that is, the coefficient of variation (CV), computed as the SD/mean value of the intertap intervals (with higher values representing lower movement regularity). Linear regression techniques were used to determine the intercept (reflecting movement amplitude in degrees and velocity in degrees/s at the beginning of the 15‐second motor sequence) and slope (representing amplitude and velocity decrement, i.e, sequence effect across the 15‐second trials) of the regression line across the scatter plot of the kinematic parameters (y‐axis) versus the number of movements (x‐axis).^32^, ^34^, ^35^, ^36^, ^37^, ^38^

### Experimental Design

Participants underwent one experimental session, including a clinical evaluation performed by a neurologist with expertise in movement disorders. All tVPA patients also underwent a kinematic evaluation of upper limb tremor.^8^, ^30^, ^31^, ^32^, ^33^ Finally, clinical and kinematic assessments of finger tapping were performed for all participants.^32^, ^34^, ^35^, ^36^, ^37^, ^38^

### Statistical Analysis

Fisher exact test was used to assess sex differences between groups. Possible differences in age or other clinical features between groups were evaluated by the Kruskal–Wallis analysis of variance (ANOVA).

Postural tremor amplitude and frequency were analyzed in tVPA patients using two separate repeated‐measure ANOVAs (rmANOVAs) with the factors “group” (2 levels: tVPA monotherapy vs. tVPA polytherapy), “posture” (2 levels: posture 1 and posture 2), and “side” (2 levels: right and left). Kinetic tremor parameters were analyzed in separate rmANOVAs using “group” (2 levels: tVPA monotherapy vs. tVPA polytherapy) and “side” (2 levels: right and left) as factors of analysis. Finally, rest tremor amplitude and frequency were analyzed in tVPA patients using separate ANOVAs with the “group” (2 levels: tVPA monotherapy vs. tVPA polytherapy) and “side” (2 levels: right and left) factors. Finger tapping kinematics in patients taking VPA (tVPA and ntVPA), noVPA patients, patients with PD, and HCs were compared by separate one‐way ANOVAs using the group factor. Fisher's least significant difference test was used for post hoc analyses. Results were corrected for multiple comparisons using the false discovery rate (FDR).^39^

Possible relationships between clinical and neurophysiological data were assessed using Spearman correlations.

All results are presented as mean values ± 1 standard error of the mean unless otherwise specified. The significance level was set at *P* < 0.05. Data were analyzed using STATISTICA (TIBCO Software Inc., Palo Alto, CA).

## Results

### Demographic and Clinical Data

We did not find any differences in terms of age, sex ratio, age at diagnosis, tremor duration, or cognitive and psychiatric scores between tVPA patients taking VPA alone and tVPA patients taking other associated antiepileptic drugs (all *P* values >0.05) (Table 2).

**TABLE 2:** Participant demographic and clinical data

Demographic and clinical data	tVPA Monotherapy	tVPA Polytherapy	ntVPA	noVPA	PD	HC
Sex	6 females/7 males	10 females/6 males	6 females/7 males	10 males/3 females	11 females/9 males	10 females/10 males
Age	50.07 ± 5.23	51.06 ± 2.68	33.84 ± 3.56	48.15 ± 4.73	57.9 ± 1.73	50.2 ± 3.6
Tremor duration	6.69 ± 1.7	8.5 ± 2.2	–	–	–	–
VPA duration	15.11 ± 3.051	14.77 ± 3.28	16.6 ± 3.98	–	–	–
MoCA	25.5 ± 0.6	26.15 ± 0.5	26.61 ± 0.44	27.92 ± 0.41	26.5 ± 0.87	27.6 ± 0.34
FAB	16 ± 1.04	15.42 ± 0.41	17.18 ± 0.4	17.38 ± 0.33	16.61 ± 0.56	17.3 ± 0.34
BDI‐II	8.92 ± 2.86	11.75 ± 3.71	8.23 ± 2.43	6.54 ± 1.71	6.55 ± 1.13	6.1 ± 1.08
FSS	28.33 ± 5.38	29.92 ± 4.49	26.3 ± 5.33	20.54 ± 2.82	26.5 ± 3.26	21.8 ± 3.85
MDS‐UPDRS III	12.91 ± 2.37	15.42 ± 2.54	1.84 ± 0.35	0.85 ± 0.34	23.6 ± 1.83	–
VPA serum concentration	70.3 ± 3.88	82.01 ± 3.83	56.68 ± 6.21	–	–	–

*Note*: Tremor duration and VPA duration are expressed in years. Data are shown as mean ± standard error of the mean. Age is expressed in years. Valproate serum concentration is expressed in μg/mL.

Abbreviations: tVPA, VPA and tremor; ntVPA, VPA without tremor; noVPA, patients with epilepsy who were not taking nor had never taken VPA; PD, Parkinson's disease; HCs, healthy controls; VPA, valproate; MoCA, Montreal Cognitive Assessment; FAB, Frontal Assessment Battery; BDI‐II, Beck Depression Inventory; FSS, Fatigue Severity Scale; MDS‐UPDRS III, Movement Disorder Society–sponsored revision of the Unified Parkinson's Disease Rating Scale, Part III.

Patients with epilepsy did not differ from patients with PD and HCs in terms of sex ratio and age, with the exception of ntVPA patients who were younger than the other groups (all *P* values <0.05) (Table 2). VPA serum concentration was similar in tVPA patients (*P* = 0.08), whereas it was lower in ntVPA compared with tVPA taking other associated antiepileptic drugs (tVPA monotherapy vs. ntVPA: *P* = 0.05; tVPA polytherapy vs. ntVPA: *P* < 0.001) (Table 2).

Clinical evaluation showed that all tVPA patients had postural tremor of the upper limbs (Table 3). The proportion of patients with kinetic and rest upper limb tremor did not differ between tVPA patients taking VPA alone and those taking VPA plus other antiepileptics. In addition, tremor affecting other body parts, including the lower limbs, head, and tongue/voice, was observed in comparable percentages in the two tVPA groups (Table 3). However, tremor was more severe in tVPA patients taking VPA plus other antiepileptics than in tVPA patients taking VPA alone, as evidenced by FTMTRS total scores (*P* = 0.03) (Table 3). This difference was attributed to higher scores obtained by tVPA patients on polytherapy on subsection B (action tremor severity) (*P* = 0.010) and subsection C (tremor disability) (*P* = 0.019), whereas similar subsection A scores were observed in the 2 tVPA patient groups (*P* = 0.182) (Table 3). Notably, none of the noVPA patients had clinically detectable tremor.

**TABLE 3:** Tremor clinical features in tVPA patients

Tremor features	tVPA Monotherapy	tVPA Polytherapy
Upper limb postural tremor	100	100
Upper limb kinetic tremor	69.23	93.75
Upper limb rest tremor	69.2	50
Head tremor	15.38	43.5
Lower limb tremor	23.07	37.5
Tongue/voice tremor	84.61	56.25
FTMTRS	19.9 ± 2.42	30.5 ± 3.0
FTMTRS‐A	8.15 ± 1.21	10.93 ± 1.54
FTMTRS‐B	7.61 ± 0.63	11.18 ± 1.50
FTMTRS‐C	3.46 ± 0.95	7.5 ± 0.92

*Note*: Data are shown as percentage or mean ± standard error of the mean.

Abbreviations: tVPA, valproate and tremor; FTMTRS, Fahn‐Tolosa‐Marin Tremor Rating Scale.

Clinical assessment revealed comparable MDS‐UPDRS III scores between tVPA patients taking VPA alone and those on polytherapy (*P* = 0.3) (Table 2). Scores were mainly influenced by the presence of tremor in patients. MDS‐UPDRS III scores were instead lower in ntVPA patients because of the absence of tremor, and near to the 0 (ranging from 0 to 3, for the presence of mild gait and posture impairment) in the noVPA group. As expected, MDS‐UPDRS III scores were higher in patients with PD compared with the other groups (all *P* values <0.05).

### Kinematic Tremor Features in tVPA

**FIG. 1:**
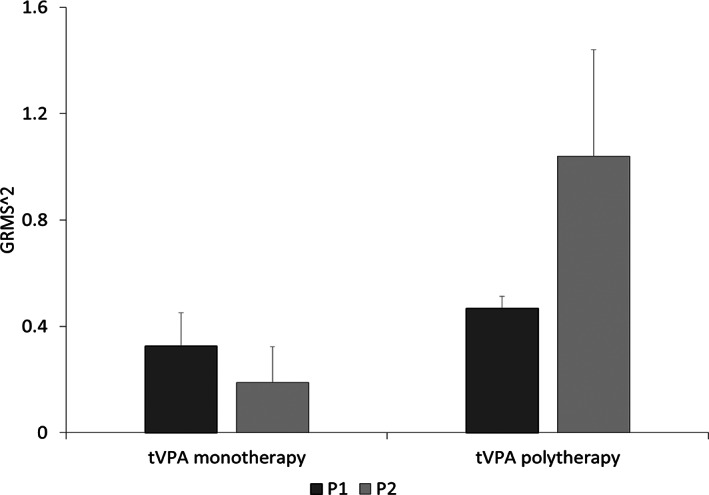
Kinematic assessment of postural upper limb tremor in patients with valproate and tremor (tVPA) taking VPA alone (tVPA monotherapy) and patients taking other antiepileptics in addition to VPA (tVPA polytherapy). Postural tremor was recorded with arms outstretched in front of the chest, that is, posture 1 (P1; dark gray), and with arms flexed at the elbows, that is, lateral “wing beating” posture or posture 2 (P2; light gray). Bars indicate tremor amplitude expressed by the root mean square of the acceleration traces (GRMS^2^) of the reference marker in three‐dimensional space. Error bars denote standard errors. Note that tremor amplitude was lower in tVPA monotherapy compared with tVPA polytherapy, particularly during P2.

Analysis of postural tremor amplitude in tVPA patients showed a significant effect of the factor “group” (*F*_1,27_ = 5.22, *P* = 0.03) and a significant “group” × “posture” interaction (*F*_1,27_ = 4.81, *P* = 0.037). Post hoc comparisons showed lower tremor amplitude in tVPA patients taking VPA alone than in tVPA patients on polytherapy during posture 2 (Fig. 1). Postural tremor frequency was instead similar in the 2 tVPA groups (Table 4), as demonstrated by rmANOVA, which did not show any significant effect of the factors or any significant interaction (“group”: *F*_1,27_ = 3.34, *P* = 0.08; “posture”: *F*_1,27_ = 1.8, *P* = 0.19; “side”: *F*_1,27_ = 0.2, *P* = 0.65; “group” × “posture”: *F*_1,27_ = 0.21, *P* = 0.64; “group” × “side”: *F*_1,27_ = 0.14, *P* = 0.71; “posture” × “side”: *F*_1,27_ = 0.75, *P* = 0.39; “group” × “posture” × “side”: *F*_1,27_ = 0.5, *P* = 0.48). Analysis of upper limb kinetic tremor did not display any differences between the two groups of tVPA patients (all *P* values >0.05) (Table 5). The ANOVA on rest tremor amplitude did not show any significant effect of the factors “group” (*F*_1,27_ = 1.78, *P* = 0.19) or “side” (*F*_1,27_ = 0.14, *P* = 0.7), nor any significant “group” × “side” interaction (*F*_1,27_ = 0.79, *P* = 0.38). Finally, rest tremor frequency was similar in the 2 tVPA groups (Table 4), as demonstrated by ANOVA that, again, did not show any significant effect of the factors “group” (*F*_1,27_ = 0.08, *P* = 0.77) or “side” (*F*_1,27_ = 1.71, *P* = 0.2), nor any significant “group” × “side” interaction (*F*_1,27_ = 2.54, *P* = 0.12).

**TABLE 4:** Postural and rest tremor frequencies in tVPA patients

Position	tVPA Monotherapy	tVPA Polytherapy
P1	5.77 ± 0.25	5.46 ± 0.14
P2	6.27 ± 0.36	5.7 ± 0.26
Rest	5.9 ± 0.29	5.64 ± 0.54

*Note*: Tremor frequencies are expressed in Hz (mean from the right and left side). Data are shown as mean ± standard error of the mean.

Abbreviations: tVPA, valproate and tremor; P1, posture 1 (arms outstretched in front of the chest); P2, posture 2 (arms flexed at the elbows, i.e., lateral “wing beating” posture).

**TABLE 5:** Kinematic parameters of kinetic tremor in tVPA patients

Parameter	tVPA Monotherapy	tVPA Polytherapy
Distance	0.42 ± 0.02	0.42 ± 0.03
N. Mov	8.65 ± 0.74	7.55 ± 0.59
PV	1.26 ± 0.09	1.18 ± 0.1
AP	11.11 ± 1.47	10.81 ± 1.65
AD	0.63 ± 0.07	0.70 ± 0.06
DD	0.31 ± 0.02	0.37 ± 0.03
D/A ratio	0.53 ± 0.04	0.54 ± 0.03
CI	1.05 ± 0.01	1.06 ± 0.01

*Note*: Shown are the means ± standard error of the mean between the right and left side of the kinematic parameters from the pointing task for kinetic tremor assessment in tVPA. Distance is expressed in meters.

Abbreviations: tVPA, valproate and tremor; N. Mov, number of movements; PV, peak of velocity, expressed in m/s; AP, acceleration peak, expressed in m/s^2^; AD, acceleration duration, expressed in minutes; DD, acceleration duration, expressed in minutes; D/A, deceleration/acceleration ratio; CI, curvature index.

In summary, confirming clinical observations, kinematic analysis showed that tVPA patients taking VPA alone had less severe postural tremor than tVPA patients taking VPA and other antiepileptic drugs.

### Kinematic Assessment of Finger Tapping in Patients Taking VPA

**FIG. 2:**
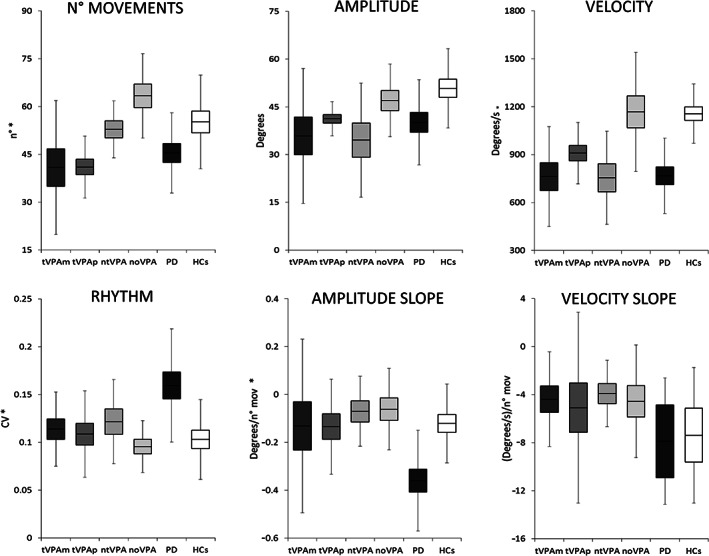
Kinematic variables of repetitive finger movements in patients with valproate (VPA) and tremor (tVPA) taking valproate alone (tVPA monotherapy), tVPA patients taking other antiepileptics in addition to VPA (tVPA polytherapy), patients taking VPA without tremor (ntVPA), patients with epilepsy who were not taking nor had never taken VPA (noVPA), patients with Parkinson's disease (PD), and healthy controls (HCs). Data were compared by a one‐way analysis of variance. Fisher test was used for post hoc comparisons. Horizontal lines denote average values. Boxes contain the mean value ± 1 standard error of the mean. Whiskers contain the mean value ± 1 standard deviation of the mean. Asterisks indicate *P* < 0.01 (corrected α level by false discovery rate). Note that at post hoc comparisons tVPA and patients with PD performed less movements compared with ntVPA, noVPA patients and HCs. Movement velocity was lower in patients taking VPA and in patients with PD than in noVPA patients and HCs, with no differences between patient groups. CV and amplitude slope values were higher in patients with PD compared with the other groups, whereas no differences in terms of these movement parameters were observed between groups of patients with epilepsy or between patients with epilepsy and HCs. Finally, note that motor performance in noVPA patients was normal. CV, coefficient of variation; N. mov, number of movements.

Analysis showed altered motor performance in patients compared with HCs, except for noVPA patients who showed normal finger tapping parameters (Fig. 2 and Video 1). In detail, 1‐way ANOVA, with 0.01 corrected α level by FDR, displayed differences between groups in terms of the number of movements (*F*_5,89_ = 6.59, *P* < 0.001), CV (*F*_5,89_ = 4.74, *P* < 0.001), movement velocity (*F*_5,89_ = 7.90, *P* < 0.001), and amplitude slope (*F*_5,89_ = 4.85, *P* < 0.001). Post hoc comparisons showed that tVPA patients and patients with PD performed less movements than HCs, ntVPA and noVPA patients (tVPA patients on monotherapy vs. HCs: *P* = 0.001; tVPA patients on polytherapy vs. HCs: *P* = 0.003; tVPA patients on monotherapy vs. noVPA: *P* < 0.001; tVPA patients on polytherapy vs. noVPA: *P* < 0.001; tVPA patients on monotherapy vs. ntVPA: *P* = 0.01; tVPA patients on polytherapy vs. ntVPA: *P* = 0.02; patients with PD vs. HCs: *P* = 0.03; patients with PD vs. noVPA: *P* < 0.001; ntVPA patients vs. HCs: *P* = 0.65; ntVPA patients vs. noVPA: *P* = 0.06; ntVPA patients vs. patients with PD: *P* = 0.13; noVPA vs. HCs: *P* = 0.1). Movement velocity was lower in patients than in HCs and noVPA (all *P* < 0.01), with no differences between groups. Conversely, CV and amplitude slope values were higher in PD compared with the other groups (all *P* < 0.01), whereas no differences in terms of these movement parameters were observed between the groups of patients with epilepsy or between patients and HCs.

**Video 1:** The video shows representative examples of finger tapping performed by the patients enrolled in the study. Segment 1 represents the finger tapping performance of one patient with valproate (VPA) and tremor taking VPA alone (tVPA monotherapy [tVPAm]), and segment 2 represents finger tapping in a patient with tremor taking other antiepileptics in addition to VPA (tVPA polytherapy [tVPAp]). Segment 3 represents the movement performance of one patient taking VPA but without tremor (ntVPA). Segment 4 represents finger tapping in one patient with epilepsy who was not on VPA treatment (and who had never been treated with VPA) (noVPA). Segment 5 represents a patient with Parkinson's disease (PD). Finally, segment 6 represents one healthy control (HC). The left panels show the video recordings. The top right panels represent the finger tapping during the kinematic recordings. Finally, the bottom right panels represent the velocity curves of the finger tapping (the y‐axis indicates the movement velocity, with values ranging from −1500 to 1500 degrees/s, and the x‐axis indicates the 15‐second recording). Note the slow motor performance in tVPAm, tVPAp, ntVPA, and PD and the normal motor performance in HC and noVPA. These videos demonstrate the importance of kinematic motion analysis to objectively assess the parameters that can be difficult to evaluate by clinical examination alone.

In summary, kinematic analysis of finger tapping showed that patients taking VPA, including ntVPA patients, performed slowed movements compared with HCs and noVPA patients. However, unlike patients with PD, they did not show sequence effect (i.e., amplitude or velocity decrement as movements were continued) or altered movement rhythm during the finger tapping performance. No differences in terms of movement velocity emerged when comparing tVPA patients taking VPA alone and those taking VPA plus other antiepileptics. Finally, noVPA patients had a normal motor performance.

### Correlations

We found a correlation between VPA serum concentration and right kinetic tremor severity as measured by the CI (*R* = 0.46, *P* = 0.012) and the D/A ratio (*R* = 0.45, *P* = 0.013). No other correlations between clinical, demographic, and kinematic data emerged from the analysis.

## Discussion

In the present article, we used clinical and kinematic analyses to investigate tremor features in patients with epilepsy taking VPA and to understand the possible effects of VPA polytherapy on tremor features. We also investigated possible voluntary movement abnormalities in patients taking VPA and their relationship with tremor. To this aim, we compared tVPA patients with patients taking VPA who had not developed tremor to exclude the possible confounding effect of tremor on motor performance. In addition, to further demonstrate that possible abnormalities of voluntary movement were attributed to VPA rather than epilepsy, we also studied patients with epilepsy who were not on VPA treatment (and who had never been treated with VPA). We found that VIT in tVPA was exacerbated by VPA association with other antiepileptic drugs. Then we observed that patients taking VPA regardless of the presence of tremor were slow but without sequence effect. Finally, we found a normal motor performance in patients with epilepsy who were not on VPA treatment, thus supporting the evidence that VPA itself, and not epilepsy, induces movement slowness in patients.

Because we did not find any differences in terms of sex distribution between groups, we could exclude this as a potential confounding factor. Our samples were all age matched, except for ntVPA patients, who were younger than the other groups. In addition, we could rule out differences between groups due to cognitive and psychiatric factors because clinical scores were comparable between groups. Because patient samples were homogeneous in terms of epileptic syndrome diagnosis, we could also exclude any confounding factors attributed to differences in epileptic syndromes. Notably, we did not perform genetic or electrophysiological tests to exclude familial cortical myoclonic tremor with epilepsy or benign adult familial myoclonic epilepsy,^40^ pathological conditions that are also characterized by rhythmic myoclonus‐resembling tremor. However, none of the patients enrolled in the study had clinical features compatible with the aforementioned diagnoses. Also, the age at epileptic syndrome onset in our patients was not suggestive of these diagnoses. Finally, we could exclude that tremor influenced upper limb motor execution because we tested finger tapping movements, which, unlike proximal arm movements, are less likely to slow down as a compensatory strategy for tremor.^19^

The first novel finding of the present study is the demonstration that tremor in patients taking VPA alone was less severe than tremor in patients taking VPA plus other antiepileptic drugs. Namely, we found that tVPA patients with VPA only had less severe action tremor as well as lower scores on subsection C of the FTMTRS, that is, a lower influence of tremor on daily activities, compared with tVPA patients on polytherapy. Kinematic assessment confirmed the clinical results, showing that postural upper limb tremor amplitude was lower in tVPA patients taking VPA alone than in tVPA patients on polytherapy. In addition, correlation analysis performed in patients taking VPA (including both monotherapy and polytherapy) showed a positive relationship between VPA serum concentration and tremor severity. Overall, these results demonstrate that VPA induces tremor when it is taken alone and that other antiepileptics worsen tremor.^2^, ^9^ Notably, no previous studies have used objective techniques to specifically investigate possible effects on tremor when other antiepileptics were used in combination with VPA. The observation that other antiepileptic drugs may exacerbate tremor in patients taking VPA was mainly based on clinical case reports and series,^11^, ^12^ which have not always reported consistent results,^3^, ^13^ and was not supported by neurophysiological evidence. The hypothesis that other antiepileptic drugs, when added to VPA, may determine the worsening of hepatic metabolism, thus causing higher plasmatic VPA levels and tremor worsening, is unlikely in our patients because serum VPA concentration was comparable in tVPA patients taking VPA alone and in those taking VPA plus other drugs. Understanding the mechanisms underlying tremor worsening in patients taking VPA plus other antiepileptics, however, is outside the scope of the present study. Our results have important clinical implications. First, they indicate the need to accurately collect medication history when evaluating tVPA patients. Furthermore, these findings suggest that for tVPA patients who cannot reduce or discontinue VPA, tremor severity may be reduced by modifying other antiepileptic therapies.

The second novel finding of the study is the observation of movement slowness during the execution of finger tapping in patients taking VPA. Although scarcely investigated, clinical evidence of VPA‐induced motor abnormalities has been reported both in animal models and in humans.^2^, ^3^, ^14^, ^15^, ^41^, ^42^, ^43^ A number of case reports, case series, and systematic analyses have documented the presence of altered voluntary movement execution in the context of a VPA‐induced parkinsonian syndrome, with prevalence ranging widely between 1.4% and 75% in patients taking VPA. These studies also showed great heterogeneity with regard to clinical presentation, age of onset, VPA dose, concomitant conditions, and imaging findings. From a neurophysiological point of view, parkinsonism in patients taking VPA has been poorly investigated,^14^ and the relationship between tremor and altered voluntary movement execution in these patients is unclear. In this regard, although we found in a previous kinematic study that tVPA patients were slower than patients with ET when performing arm movements,^8^ we could not exclude the possible influence of tremor on movement slowness. In the present study, we found that all patients taking VPA, including those who had not developed tremor, performed slower finger tapping compared with HCs. This indicates that the detrimental effect of VPA on motor performance is independent of tremor occurrence. Accordingly, correlation analysis did not show any relationship between slowed motor performance and tremor severity in tVPA patients. In addition, we found that finger tapping motor performance was normal in patients with epilepsy who were not on VPA treatment (and who had never been treated with VPA), thus supporting the evidence that VPA itself induces movement slowness in patients.

A further result to be discussed is the observation that, although all tVPA patients showed movement slowness when performing finger tapping movements, not all VPA patients who showed movement slowness had developed upper limb tremor. It is well known that 6% to 45% of patients taking VPA may develop tremor within 3 to 12 months of VPA initiation.^44^ However, all patients we tested were on VPA therapy for at least 1 year prior to study enrollment. Also, because there were no differences in VPA treatment duration between tVPA and ntVPA patients, it is unlikely that ntVPA patients did not develop tremor because they started VPA more recently. Notably, VPA serum concentrations in ntVPA patients were lower than concentrations found in tVPA patients. In addition, ntVPA patients were younger than tVPA patients, suggesting that age‐related effects must also be considered in the development of tremor. Overall, these results suggest that tremor is probably not the first motor adverse effect of VPA treatment but may indicate that tremor has a higher threshold to manifest, perhaps facilitated by aging and/or higher drug concentration. In other words, movement slowness and tremor induced by VPA may emerge at different times during VPA treatment, with movement slowness starting first. The present hypothesis is also supported by findings of a previous neurophysiological study that showed that motor performance was impaired in the majority of VPA‐treated patients even if they did not show any tremor.^16^ Further longitudinal studies, however, are needed to better clarify the present issue.

Our results may be interpreted from a pathophysiological standpoint. VPA interacts with and alters gamma‐Aminobutyrc acid (GABA) transmission.^45^ In addition, it interferes with gene expression by downregulating the transcription of genes involved in synaptic plasticity and neuronal survival^46^, ^47^ and by acting as a histone deacetylase, thus unpacking DNA from histones.^48^ Finally, VPA may enhance neurodegeneration through free oxygen radicals.^49^, ^50^ In humans, however, it is not clear which of the aforementioned mechanisms is the major determinant of VPA adverse effects. It is also not clear what neural circuits are the most affected by VPA. It has been suggested that VPA has a toxic effect on the cerebellum.^51^, ^52^, ^53^, ^54^, ^55^ VPA may affect cerebellar trophism and function by reducing the number of Purkinje cells and disrupting cerebellar circuitry.^51^, ^52^, ^53^, ^54^, ^55^ Notably, the role of the cerebellum in tremor pathophysiology has been extensively demonstrated.^8^, ^30^, ^31^, ^32^, ^56^ In addition, the cerebellum is an important hub regulating several movement parameters, including speed, direction, and coordination,^57^ and cerebellar damage may result in movement slowness.^19^, ^22^, ^58^ Thus, one hypothesis is that VIT and VPA‐induced movement slowness reflect cerebellar network disruption.

However, there is also evidence that VPA administration induces a detrimental effect on the basal ganglia and alters dopaminergic transmission.^14^, ^41^, ^42^, ^59^ This effect has been related to a VPA‐induced excess of GABAergic activity in the basal ganglia, which in turn provokes a reduction in dopamine release in the substantia nigra and hyperactivity of the globus pallidus externus, resulting in unbalanced activation of indirect basal ganglia pathways.^60^, ^61^ Accordingly, it has been demonstrated that some patients with VPA‐induced parkinsonism had altered dopamine transporter (DAT) imaging.^41^ The role of the basal ganglia in motor control and in generating bradykinesia in parkinsonian conditions such as PD is well established.^19^, ^62^ However, in the present study we found that patients taking VPA had different motor features than patients with PD, that is, they did not show altered movement rhythm or, most important, the sequence effect, which is a cardinal feature of PD bradykinesia.^17^, ^18^, ^19^, ^20^, ^21^, ^63^, ^64^, ^65^, ^66^ Although we did not perform a DAT scan on our patients taking VPA, this result may suggest that VPA‐induced slowness is dependent on different mechanisms than bradykinesia in PD.

The present study has some limitations. The sample size was relatively limited, although the objective techniques used to quantify finger tapping allowed us to obtain accurate and reproducible measurements of motor impairment.^67^, ^68^ In addition, ntVPA patients were younger than the other patient groups. We did not collect serum concentrations of any antiepileptic drugs other than VPA. Also, because we did not perform a longitudinal follow‐up of the patients we studied, unanswered issues remain regarding whether the presence of movement slowness in ntVPA patients suggests that all patients will later develop tremor and/or parkinsonism or whether there are possible individual susceptibility factors. Thus, further longitudinal investigations are needed to better understand the possible progression of tremor and slowness of movement induced by VPA intake over time. A further limitation is that all patients we tested had a diagnosis of epilepsy. Testing patients treated with VPA for a nonepileptic indication (e.g., migraines, bipolar disorder, mood and anxiety disorders) may be warranted for a thorough understanding of disease‐specific vulnerability to VPA. Also, patients did not have DAT scans.

In conclusion, we objectively demonstrated that tremor and movement slowness are motor signs due to VPA intake. The severity of VIT was exacerbated by the association of VPA with other antiepileptic drugs. Movement slowness was detectable in all patients taking VPA; it occurred independent of tremor and was specifically attributed to VPA and not to epilepsy. These findings have important clinical implications because they may indicate that the appearance of neurophysiologically detectable slowness of movement may precede tremor development in patients taking VPA.

### Availability of Data and Material

Data that support the findings of this study are available upon request to the corresponding author.

## Author Roles

(1) Research Project: A. Conception, B. Organization, C. Execution; (2) Statistical Analysis: A. Design, B. Execution, C. Review and Critique; (3) Manuscript Preparation: A. Writing of the First Draft, B. Review and Critique.

A.D.B.: 1B, 1C, 2A, 2B, 3A

G.P.: 1B, 1C, 2A, 2B, 3A

L.A.: 1C, 2C, 3B

A.C.: 1C, 2C, 3B

D.C.: 1C, 2C, 3B

E.C.I.: 1B, 2C, 3B

A.T.G.: 1B, 2C, 3C

C.D.B.: 1B, 2C, 3C

A.B.: 2C, 3B

M.B.: 1A, 1B, 2C, 3B

## Disclosures

### Ethical Compliance Statement

The local ethics committee approved the study. All participants provided written informed consent to participate in the study. All authors have read the Journal's position on issues involved in ethical publication and affirm that this work is consistent with those guidelines.

### Funding Sources and Conflicts of Interest

This research did not receive any specific grant from funding agencies in the public, commercial, or not‐for‐profit sectors. None of the authors have any potential conflicts of interest to disclose.

### Financial Disclosures for the Previous 12 Months

None of the authors have any financial interests to disclose.

## References

[1] Perucca E . Pharmacological and therapeutic properties of valproate: A summary after 35 years of clinical experience. CNS Drugs 2002;16(10):695–714. 12269862 10.2165/00023210-200216100-00004

[2] Zadikoff C , Munhoz RP , Asante AN , Politzer N , Wennberg R , Carlen P , Lang A . Movement disorders in patients taking anticonvulsants. J Neurol Neurosurg Psychiatry 2007;78(2):147–151. 17012337 10.1136/jnnp.2006.100222 PMC2077655

[3] Nouzeilles M , García M , Rabinowicz A , Merello M . Prospective evaluation of parkinsonism and tremor in patients treated with valproate. Parkinsonism Relat Disord 1999;5(1–2):67–68. 18591122 10.1016/s1353-8020(99)00013-9

[4] Karas BJ , Wilder BJ , Hammond EJ , Bauman AW . Valproate tremors. Neurology 1982;32(4):428–432. 6801541 10.1212/wnl.32.4.428

[5] Hyman NM , Dennis PD , Sinclair KG . Tremor due to sodium valproate. Neurology 1979;29(8):1177–1180. 379690 10.1212/wnl.29.8.1177

[6] Rinnerthaler M , Luef G , Mueller J , et al. Computerized tremor analysis of valproate‐induced tremor: A comparative study of controlled‐release versus conventional valproate. Epilepsia 2005;46(2):320–323. 15679514 10.1111/j.0013-9580.2005.36204.x

[7] Mehndiratta MM , Satyawani M , Gupta S , Khwaja GA . Clinical and surface EMG characteristics of valproate induced tremors. Electromyogr Clin Neurophysiol 2005;45(3):177–182. 15981690

[8] Paparella G , Angelini L , De Biase A , et al. Clinical and kinematic features of valproate‐induced tremor and differences with essential tremor. Cerebellum 2021;20(3):374–383. 33200286 10.1007/s12311-020-01216-5 PMC8213593

[9] Morgan JC , Sethi KD . Drug‐induced tremors. Lancet Neurol 2005;4(12):866–876. 16297844 10.1016/S1474-4422(05)70250-7

[10] Alonso‐Juarez M , Baizabal‐Carvallo JF . Distinguishing features between valproate‐induced tremor and essential tremor. Acta Neurol Scand 2018;138(2):177–181. 29749618 10.1111/ane.12953

[11] Reutens DC , Duncan JS , Patsalos PN . Disabling tremor after lamotrigine with sodium valproate. Lancet 1993;342(8864):185–186. 10.1016/0140-6736(93)91398-6 8101290

[12] He ZF , Chen J , Zhou CN , Rao Z , Wang XH . Disabling tremor induced by long‐term use of sodium valproate and lamotrigine: Case report. Medicine 2017;96(47):e8711. 29381960 10.1097/MD.0000000000008711 PMC5708959

[13] Lan L , Zhao X , Jian S , et al. Investigation of the risk of valproic acid‐induced tremor: Clinical, neuroimaging, and genetic factors. Psychopharmacology (Berl) 2022;239(1):173–184. 34718848 10.1007/s00213-021-06004-5

[14] Jamora D , Lim SH , Pan A , Tan L , Tan EK . Valproate‐induced parkinsonism in epilepsy patients. Mov Disord 2007;22(1):130–133. 17115396 10.1002/mds.21188

[15] Baizabal‐Carvallo JF , Alonso‐Juarez M . Valproate‐induced rest tremor and parkinsonism. Acta Neurol Belg 2021;121(2):515–519. 31721077 10.1007/s13760-019-01239-8

[16] Farkas Z , Gulyás S , Molnár R , Szirmai I , Kamondi A . Quantitative analysis of motor performance in epilepsy patients treated with valproate. Seizure 2010;19(3):173–177. 20167509 10.1016/j.seizure.2010.01.013

[17] Postuma RB , Berg D , Stern M , et al. MDS clinical diagnostic criteria for Parkinson's disease. Mov Disord 2015;30(12):1591–1601. 26474316 10.1002/mds.26424

[18] Berardelli A , Wenning GK , Antonini A , et al. EFNS/MDS‐ES/ENS [corrected] recommendations for the diagnosis of Parkinson's disease. Eur J Neurol 2013;20(1):16–34. 23279440 10.1111/ene.12022

[19] Bologna M , Paparella G , Fasano A , Hallett M , Berardelli A . Evolving concepts on bradykinesia. Brain 2020;143(3):727–750. 31834375 10.1093/brain/awz344 PMC8205506

[20] Goetz CG , Tilley BC , Shaftman SR , et al. Movement Disorder Society‐sponsored revision of the unified Parkinson's disease rating scale (MDS‐UPDRS): Scale presentation and clinimetric testing results. Mov Disord 2008;23(15):2129–2170. 19025984 10.1002/mds.22340

[21] Antonini A , Abbruzzese G , Ferini‐Strambi L , Tilley B , Huang J , Stebbins GT , et al. Validation of the Italian version of the Movement Disorder Society—Unified Parkinson's disease rating scale. Neurol Sci 2013;34(5):683–687. 22678179 10.1007/s10072-012-1112-z

[22] Paparella G , Fasano A , Hallett M , Berardelli A , Bologna M . Emerging concepts on bradykinesia in non‐parkinsonian conditions. Eur J Neurol 2021;28:2403–2422. 33793037 10.1111/ene.14851

[23] Fisher RS , Cross JH , French JA , et al. Operational classification of seizure types by the international league against epilepsy: Position paper of the ILAE Commission for Classification and Terminology. Epilepsia 2017;58(4):522–530. 28276060 10.1111/epi.13670

[24] Hoehn MM , Yahr MD . Parkinsonism: Onset, progression, and mortality. Neurology 1967;17(5):427. 6067254 10.1212/wnl.17.5.427

[25] Nasreddine ZS , Phillips NA , Bédirian V , et al. The Montreal cognitive assessment, MoCA: A brief screening tool for mild cognitive impairment. J Am Geriatr Soc 2005;53(4):695–699. 15817019 10.1111/j.1532-5415.2005.53221.x

[26] Dubois B , Slachevsky A , Litvan I , Pillon B . The FAB: A frontal assessment battery at bedside. Neurology 2000;55(11):1621–1626. 11113214 10.1212/wnl.55.11.1621

[27] Beck AT , Ward CH , Mendelson M , Mock J , Erbaugh J . An inventory for measuring depression. Arch Gen Psychiatry 1961;4:561–571. 13688369 10.1001/archpsyc.1961.01710120031004

[28] Fahn S , Tolosa E , Marín C . Clinical rating scale for tremor. In: Jankovik J. and Tolosa E. Parkinson's disease and movement disorders. Baltimore‐Munich: Urban & Schwarzenberg; 1988:225–234.

[29] Friedman JH , Alves G , Hagell P , et al. Fatigue rating scales critique and recommendations by the movement disorders society task force on rating scales for Parkinson's disease. Mov Disord 2010;25(7):805–822. 20461797 10.1002/mds.22989

[30] Bologna M , Berardelli I , Paparella G , et al. Tremor distribution and the variable clinical presentation of essential tremor. Cerebellum 2019;18(5):866–872. 31422549 10.1007/s12311-019-01070-0

[31] Paparella G , Ferrazzano G , Cannavacciuolo A , Cogliati Dezza F , Fabbrini G , Bologna M , Berardelli A . Differential effects of propranolol on head and upper limb tremor in patients with essential tremor and dystonia. J Neurol 2018;12:2695–2703. 10.1007/s00415-018-9052-z 30209649

[32] Bologna M , Paparella G , Colella D , et al. Is there evidence of bradykinesia in essential tremor? Eur J Neurol 2020;27:1501–1509. 32396976 10.1111/ene.14312

[33] Passaretti M , De Biase A , Paparella G , et al. Worsening of essential tremor after SARS‐CoV‐2 infection. Cerebellum 2022;6:1‐4. 10.1007/s12311-022-01366-8 PMC8732967 34989982

[34] Bologna M , Leodori G , Stirpe P , et al. Bradykinesia in early and advanced Parkinson's disease. J Neurol Sci 2016;15(369):286–291. 10.1016/j.jns.2016.08.028 27653910

[35] Bologna M , Guerra A , Paparella G , et al. Neurophysiological correlates of bradykinesia in Parkinson's disease. Brain 2018;141(8):2432–2444. 29901693 10.1093/brain/awy155

[36] Paparella G , Ceccanti M , Colella D , et al. Bradykinesia in motoneuron diseases. Clin Neurophysiol 2021;132(10):2558–2566. 34479133 10.1016/j.clinph.2021.08.006

[37] Bologna M , Guerra A , Colella D , et al. Bradykinesia in Alzheimer's disease and its neurophysiological substrates. Clin Neurophysiol 2020;131(4):850–858. 32066104 10.1016/j.clinph.2019.12.413

[38] Colella D , Guerra A , Paparella G , et al. Motor dysfunction in mild cognitive impairment as tested by kinematic analysis and transcranial magnetic stimulation. Clin Neurophysiol 2021;132(2):315–322. 33450553 10.1016/j.clinph.2020.10.028

[39] Curran‐Everett D . Multiple comparisons: Philosophies and illustrations. Am J Physiol Regul Integr Comp Physiol 2000;279(1):R1–R8. 10896857 10.1152/ajpregu.2000.279.1.R1

[40] Striano P , de Falco FA , Minetti C , Zara F . Familial benign nonprogressive myoclonic epilepsies. Epilepsia 2009;50(Suppl 5):37–40. 10.1111/j.1528-1167.2009.02118.x 19469844

[41] Brugger F , Bhatia KP , Besag FMC . Valproate‐associated parkinsonism: A critical review of the literature. CNS Drugs 2016;30(6):527–540. 27255404 10.1007/s40263-016-0341-8

[42] Vamos E , Csati A , Vecsei L , Klivenyi P . Effects of valproate on the dopaminergic system in mice. Neurol Res 2009;31(3):217–219. 18768113 10.1179/174313208X346099

[43] Mychasiuk R , Richards S , Nakahashi A , Kolb B , Gibb R . Effects of rat prenatal exposure to valproic acid on behaviour and neuro‐anatomy. Dev Neurosci 2012;34(2–3):268–276. 22890088 10.1159/000341786

[44] Arbaizar B , Gómez‐Acebo I , Llorca J . Postural induced‐tremor in psychiatry. Psychiatry Clin Neurosci 2008;62(6):638–645. 19067999 10.1111/j.1440-1819.2008.01877.x

[45] Löscher W . Basic pharmacology of valproate: A review after 35 years of clinical use for the treatment of epilepsy. CNS Drugs 2002;16(10):669–694. 12269861 10.2165/00023210-200216100-00003

[46] Yuan PX , Huang LD , Jiang YM , Gutkind JS , Manji HK , Chen G . The mood stabilizer valproic acid activates mitogen‐activated protein kinases and promotes neurite growth. J Biol Chem 2001;276(34):31674–31683. 11418608 10.1074/jbc.M104309200

[47] Chen G , Manji HK . The extracellular signal‐regulated kinase pathway: An emerging promising target for mood stabilizers. Curr Opin Psychiatry 2006;19(3):313–323. 16612219 10.1097/01.yco.0000218604.63463.cd

[48] Johannessen CU . Mechanisms of action of valproate: A commentatory. Neurochem Int 2000;37(2–3):103–110. 10812195 10.1016/s0197-0186(00)00013-9

[49] Salimi A , Alyan N , Akbari N , Jamali Z , Pourahmad J . Selenium and L‐carnitine protects from valproic acid‐induced oxidative stress and mitochondrial damages in rat cortical neurons. Drug Chem Toxicol 2020;4:1–8. 10.1080/01480545.2020.1810259 32885679

[50] Chaudhary S , Sahu U , Parvez S . Melatonin attenuates branch chain fatty acid induced apoptosis mediated neurodegeneration. Environ Toxicol 2021;36(4):491–505. 33219756 10.1002/tox.23055

[51] Main SL , Kulesza RJ . Repeated prenatal exposure to valproic acid results in cerebellar hypoplasia and ataxia. Neuroscience 2017;6(340):34–47. 10.1016/j.neuroscience.2016.10.052 27984183

[52] Papazian O , Cañizales E , Alfonso I , Archila R , Duchowny M , Aicardi J . Reversible dementia and apparent brain atrophy during valproate therapy. Ann Neurol 1995;38(4):687–691. 7574471 10.1002/ana.410380423

[53] Ghosh VB , Kapoor S , Prakash A , Bhatt S . Cerebellar atrophy in a child with valproate toxicity. Indian J Pediatr 2011;78(8):999–1001. 21243535 10.1007/s12098-010-0332-6

[54] Spisák T , Román V , Papp E , et al. Purkinje cell number‐correlated cerebrocerebellar circuit anomaly in the valproate model of autism. Sci Rep 2019;9(1):9225. 31239528 10.1038/s41598-019-45667-1 PMC6592903

[55] Gifford JJ , Deshpande P , Mehta P , Wagner GC , Kusnecov AW . The effect of Valproic acid exposure throughout development on microglia number in the prefrontal cortex, Hippocampus and Cerebellum. Neuroscience 2022;481:166–177. 34780921 10.1016/j.neuroscience.2021.11.012

[56] Haubenberger D , Hallett M . Essential Tremor. N Engl J Med 2018;379(6):595–597. 10.1056/NEJMc1807690 30089076

[57] Ebner TJ . A role for the cerebellum in the control of limb movement velocity. Curr Opin Neurobiol 1998;8(6):762–769. 9914240 10.1016/s0959-4388(98)80119-0

[58] Hallett M , Berardelli A , Matheson J , Rothwell J , Marsden CD . Physiological analysis of simple rapid movements in patients with cerebellar deficits. J Neurol Neurosurg Psychiatry 1991;54(2):124–133. 2019837 10.1136/jnnp.54.2.124 PMC1014346

[59] Silver M , Factor SA . Valproic acid‐induced parkinsonism: Levodopa responsiveness with dyskinesia. Parkinsonism Relat Disord 2013;19(8):758–760. 23632325 10.1016/j.parkreldis.2013.03.016

[60] Paladini CA , Celada P , Tepper JM . Striatal, pallidal, and pars reticulata evoked inhibition of nigrostriatal dopaminergic neurons is mediated by GABA(a) receptors in vivo. Neuroscience 1999;89(3):799–812. 10199614 10.1016/s0306-4522(98)00355-8

[61] Calabresi P , Picconi B , Tozzi A , Ghiglieri V , Di Filippo M . Direct and indirect pathways of basal ganglia: A critical reappraisal. Nat Neurosci 2014;17(8):1022–1030. 25065439 10.1038/nn.3743

[62] Berardelli A , Rothwell JC , Thompson PD , Hallett M . Pathophysiology of bradykinesia in Parkinson's disease. Brain 2001;124(Pt 11):2131–2146. 11673316 10.1093/brain/124.11.2131

[63] Agostino R , Currà A , Giovannelli M , Modugno N , Manfredi M , Berardelli A . Impairment of individual finger movements in Parkinson's disease. Mov Disord 2003;18(5):560–565. 12722170 10.1002/mds.10313

[64] Agostino R , Berardelli A , Formica A , Accornero N , Manfredi M . Sequential arm movements in patients with Parkinson's disease, Huntington's disease and dystonia. Brain 1992;115(Pt 5):1481–1495. 1422799 10.1093/brain/115.5.1481

[65] Espay AJ , Beaton DE , Morgante F , Gunraj CA , Lang AE , Chen R . Impairments of speed and amplitude of movement in Parkinson's disease: A pilot study. Mov Disord 2009;24(7):1001–1008. 19230031 10.1002/mds.22480

[66] Espay AJ , Giuffrida JP , Chen R , et al. Differential response of speed, amplitude, and rhythm to dopaminergic medications in Parkinson's disease. Mov Disord 2011;26(14):2504–2508. 21953789 10.1002/mds.23893 PMC3318914

[67] Heldman DA , Giuffrida JP , Chen R , et al. The modified bradykinesia rating scale for Parkinson's disease: Reliability and comparison with kinematic measures. Mov Disord 2011;26(10):1859–1863. 21538531 10.1002/mds.23740 PMC3324112

[68] Heldman DA , Espay AJ , LeWitt PA , Giuffrida JP . Clinician versus machine: Reliability and responsiveness of motor endpoints in Parkinson's disease. Parkinsonism Relat Disord 2014;20(6):590–595. 24661464 10.1016/j.parkreldis.2014.02.022 PMC4028404

